# On Mobility Management in Multi-Sink Sensor Networks for Geocasting of Queries

**DOI:** 10.3390/s111211415

**Published:** 2011-12-01

**Authors:** Ayşegül Tüysüz Erman, Arta Dilo, Lodewijk van Hoesel, Paul Havinga

**Affiliations:** 1 Pervasive Systems Research Group, Department of Computer Science, University of Twente, P.O. Box 217, 7500 AE, Enschede, The Netherlands; E-Mails: a.dilo@utwente.nl (A.D.); p.j.m.havinga@utwente.nl (P.H.); 2 Ambient Systems B.V., Colosseum 15d, 7521 PV, Enschede, The Netherlands; E-Mail: lodewijk.vanhoesel@ambient-systems.net

**Keywords:** geocasting, convex hull, local updating, mobile sensors, wireless sensor networks

## Abstract

In order to efficiently deal with location dependent messages in multi-sink wireless sensor networks (WSNs), it is key that the network informs sinks what geographical area is covered by which sink. The sinks are then able to efficiently route messages which are only valid in particular regions of the deployment. In our previous work (see the 5th and 6th cited documents), we proposed a combined coverage area reporting and geographical routing protocol for location dependent messages, for example, queries that are injected by sinks. In this paper, we study the case where we have static sinks and mobile sensor nodes in the network. To provide up-to-date coverage areas to sinks, we focus on handling node mobility in the network. We discuss what is a better method for updating the routing structure (*i.e.*, routing trees and coverage areas) to handle mobility efficiently: periodic *global updates* initiated from sinks or *local updates* triggered by mobile sensors. Simulation results show that local updating perform very well in terms of query delivery ratio. Local updating has a better scalability to increasing network size. It is also more energy efficient than our previously proposed approach, where global updating in networks have medium mobility rate and speed.

## 1. Introduction

Wireless Sensor Networks (WSNs) are a specific class of *ad hoc* networks composed of many tiny devices called sensor nodes spread throughout a given area and capable of collecting information about the surrounding environment, of processing this information, and of circulating it through the network via a wireless communication channel. Applications of WSNs have emerged in many domains ranging from environmental monitoring to industrial automation as well as disaster management. In all these applications, one of the fundamental operations in a wireless sensor network is convergecast, which refers to the communication pattern where data is collected from a set of sensor nodes and forwarded towards a common end-point gateway, namely a *sink* node, in the network. Initial deployments of wireless sensor networks had one sink, but many current WSN applications require multiple sinks. Having multiple sinks in the network gives advantages such as energy efficiency, reliability and alleviation of the uneven energy depletion problem of a single-sink deployment [1].

The work presented in this paper is mainly motivated by partly stationary, multi-sink deployments of WSNs such as real-time surveillance and city pollution monitoring applications. In these scenarios, the large majority of the deployed nodes are fixed and constitute the infrastructure of the network. In addition, the presence of a fraction of mobile nodes contributes to the overall information gathering process with local and often volatile knowledge [2]. For instance, in a firefighting scenario, firefighters and small helicopters (*i.e.*, UAVs) can carry sensor nodes on board to collect data from the fire area. In city pollution monitoring, sensor nodes attached to buses collaborating with a sensor network distributed across the city would be an efficient tool to monitor pollution and presence of contaminants, both during normal city life and in case of emergency (e.g., for the detection of nuclear, chemical threats). In these large scale applications, deployment of multiple sinks is required for efficient data gathering from WSNs.

A typical way of extracting information from a sensor network is to disseminate special messages, namely *queries*, from sink nodes to sensor nodes, asking them to send data which have the properties specified in the queries. The main consideration in designing query dissemination algorithms is to efficiently forward queries from sink nodes to sensor nodes. Especially, in a multi-sink sensor network if a query is only valid in particular regions of the deployment, it is important to determine which sinks need to inject the query to reach all sensor nodes in the area of interest.

The simplest approach to deliver a message to all nodes within a geographical region (*i.e.*, geocasting) is *simple flooding* of the message from all sinks in the whole network irrespective of the destination region and coverage areas of the sinks. Since simple flooding is not an efficient approach in terms of communication overhead, another class of geocasting approaches, called *directed flooding*, has been proposed [3] to limit message overhead and network congestion by defining forwarding zone, which comprises a subset of all nodes in the network. To employ directed flooding in a multi-sink sensor network, the network should inform sinks what geographical area is covered by which sink. This requires partitioning (clustering) of the network between different sinks, which is generally performed using energy-aware or any other metric-based route selection schemes. Data collection/extraction in WSNs is often performed by using one of the fundamental approaches, tree-based routing that forms a spanning tree rooted at the sink [4]. In a multi-sink WSN, the tree-based routing combined with multi-sink partitioning forms multiple routing trees rooted at different sinks, where each sensor node in the network is connected to only one sink (e.g., the closest sink).

**Figure 1.**
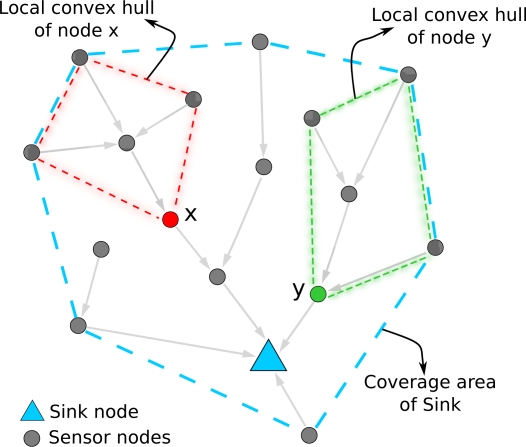
Example network with coverage area (*i.e.*, convex hull stored in the sink) and local convex hulls stored in sensor nodes.

In our previous work [5,6], we design and evaluate a geocasting protocol for query dissemination called GeoCHT, which uses forwarding zones defined by local coverage areas of sensors and sinks in a tree-based network. The local coverage area of a sensor node in a tree-based routing is made of all descendant nodes of this sensor node. Figure 1 illustrates local coverage areas of a sink and sensor nodes. In the figure, the local coverage area of a node, for instance, node x, covers all its descendants. The local coverage area of the sink covers all the nodes in the tree. The local coverage areas are used for efficient geocasting of queries from the sinks towards an area of interest. Coverage area based geocasting restricts the area in which the queries are forwarded, thus achieves energy saving in a WSN. It also directs the queries towards the region of interest, as a results decreases the query delivery delay. In our previous work, the maintenance (due to topology changes such as node failures or mobility) of the routing structure (*i.e.*, trees and coverage areas) is done by global reconstruction of the whole structure periodically. In global updating, sinks trigger the updating process by flooding special messages (called *hello* packets) over the network. Global updating forms a reliable routing structure for query dissemination. However, it may result in high energy consumption since it also updates the parts of the structure that are not affected by topology changes.

In this paper, we address the limitations and drawbacks of our previous updating approach and focus on handling topology changes due to sensor mobility. The final goal is achieving reliable geocasting of queries in multi-sink WSNs in an energy efficient way. Here, the sensor network under consideration has a hybrid network architecture composed of fixed and mobile sensor nodes. From a communication perspective, mobility of nodes can be handled either by the medium access control (MAC) protocol [7] at the data link layer or by the routing protocol in the networking layer. Cross-layered approaches [8] are also proposed for mobility management. This work focuses on handling mobility in the *network layer*, which is implemented by the *routing protocol*. The design of our geocasting protocol and mobility handling mechanism are independent of the underlying MAC protocol although we use some cross-layer information provided by MAC layer in the network layer such as localization information. After construction of the *geocast structure* (*i.e.*, routing trees plus local coverage areas of sensors and sinks in the network layer), mobile nodes move, which changes the structure of the trees and local coverage areas. In such a WSN, it is crucial to support mobility of nodes by keeping the routing trees and local coverage areas up-to-date for efficient query dissemination. Since the global updating mechanism discussed in our previous work [5,6] may be costly, there is a need for a mechanism to locally associate/re-associate mobile nodes and their child nodes to new parent nodes and update local coverage areas of sensors and sinks. In this work, we propose a mechanism, called *local updating*, which detects the changes in the neighborhood of a mobile node and updates only the parts of the geocast structure that are affected by mobility.

Managing node mobility in a tree-based forwarding scheme creates an extra overhead in the network. In this paper, the reduction of the message overhead and the overall energy consumption of the WSN is the foremost goal as well as reliability of geocasting of queries. Hence, we discuss what is a better method to handle mobility in tree-based routing of queries: Periodic *global updates* initiated by sinks or *local updates* triggered by mobile sensors. The proposed local updating mechanism utilizes *beacon* packets sent by mobile nodes to keep track of topology changes. A *beacon* packet is only forwarded to the one-hop neighbors of a mobile node in local updating; on the other hand, a *hello* packet sent by a sink is flooded all over the network in global updating. The distinctive features of the proposed local updating solution are:

1. Sending beacon packets from mobile sensor nodes instead of hello messages from every sink node,
2. The use of proactive procedures to speed up the parent-child re-association and coverage area updating phases,
3. A reduced impact of the messaging overhead to manage node mobility by resorting to local updating procedures.

In the remainder of this paper, we first discuss the related works in Section 2. For completeness, in Section 3 we briefly explain our previous work, the local coverage area based geocasting of queries and how we construct and maintain local coverage areas with the global updating mechanism. Section 4 gives the details of our new method, *local updating* mechanism. We evaluate the performance of global and local updating mechanism in Section 5. To compare global and local updating mechanism, we test their performances in terms of routing accuracy and communication overhead for different scenarios where we vary the network size to see the scalability of the methods, the mobility rate (*i.e.*, number of mobile nodes) and the mobility speed to see the effects of mobility on the performance of the methods. Finally, Section 6 draws the conclusions.

## 2. Related Work

There are a number of related approaches in the area of query dissemination, geocasting and multi-sink partitioning in wireless sensor networks. In what follows we discuss some well known approaches briefly.

### 2.1. Query Dissemination

A common approach for disseminating query messages in a WSN is a flooding mechanism, which requires any intermediate receiver to rebroadcast a non-duplicated interest packet to all its neighbors. For example, in directed diffusion [9] one of the well-known query-based routing protocols in wireless sensor networks, a sink node initiates dissemination of an interest packet throughout the entire network by flooding. A node receiving the interest sets up a gradient which indicates from whom this interest message has previously been forwarded. Although some additional features such as a gradient reinforcement have been proposed, the directed diffusion with such a flooding of interest messages obviously increases network traffic and leads to inefficient energy consumption on sensor nodes. There are some other proposals for query dissemination in WSNs such as minimum broadcast tree algorithms [10] or epidemic approaches like gossiping [11]. In this paper, the presented approach differs from the discussed approaches by using the position information of the sensors to form sensor node’s coverage area. The coverage areas [5] are used to scope the query dissemination between sinks and the target region.

### 2.2. Geographical Routing and Geocasting

A different routing strategy for wireless sensor networks is described in [12]: geographical routing. Instead of advertising an interest for data, or requesting to establish a route to a certain destination device, nodes use a routing technique based on node coordinates. Nodes are assumed to know their own position and the position of the destination node (*i.e.*, the node where the message needs to be delivered). The idea is that nodes advertise data along with the coordinates where it must be delivered. Nodes closer to the destination node consider themselves candidates for relaying the message. In most of the geographical routing protocols such as Greedy Perimeter Stateless Routing (GPSR) [13], the packets are sent from source to a destination position. GPSR is a geographic routing protocol for wireless networks that works in two modes: greedy mode and perimeter mode. In greedy mode each node forwards the packet to the neighbor closest to the destination. When greedy forwarding is not possible (*i.e.*, local maxima may exist where no neighbor is closer to the destination), the packet switches to perimeter mode, where perimeter routing (face routing [14,15]) is used to route around dead-ends (with right-hand rule [16]) until closer nodes to the destination are found.

All geographic routing research to date has assumed that for face routing to work, the underlying network graph must be made *planar* by selecting a subset of the graph’s edges. Note that a planar graph consists of faces, enclosed polygonal regions bounded by edges. Although practical distributed *planarization* is now a solved problem, due to the high communication/maintenance costs and complexities associated with the deployment of face routing algorithms, alternative approaches to face routing have been proposed. Greedy Distributed Spanning Tree Routing (GDSTR) [17] algorithm, which does not require planarization, builds a *hull tree* for forwarding packets between two sensor nodes: the root of each subtree maintains the convex hull of the locations of all descendants, as well as the convex hull of all children. In addition, each node maintains the list of hulls that intersect with its own. When greedy routing encounters a dead-end, packets are forwarded up the hull tree until they reach a node whose hull tree encloses the destination. At that point, the packet is forwarded down the tree.

For some other scenarios like general position-based publish-and-subscribe services, it is also sufficient for some packets (e.g., queries) to reach any destination currently located in a given area, which is called *geocasting*. Yu *et al.* propose Geographical and Energy-Aware Routing (GEAR) algorithm [18], which shows how to broadcast a message to all the nodes in a target region. GEAR uses greedy forwarding to forward packets to the nodes that are progressively closer to the centroid of the target region, whilst trying to balance the energy consumption at the intermediate nodes. Once the message is delivered to the centroid of the target region, it uses restricted flooding, namely Recursive Geographic Forwarding, to broadcast the message to all remaining nodes in the given region. Instead of using geographical forwarding, GeoTORA [19] uses a unicast (*ad-hoc*) routing protocol (TORA [20]) to deliver the packet to the region and then floods within the region. GEAR and GeoTORA protocols are non-flooding based approaches in which other routing protocols (*i.e.*, greedy forwarding, *ad-hoc* routing) are used instead of flooding to reach the target region of a geocast.

The authors in [3] also discuss *directed flooding* based geocast routing protocols. Directed flooding tries to limit the message overhead and network congestion of naive flooding by defining a forwarding zone, which consists of a subset of all network nodes. The forwarding zone (e.g., rectangle, cone) includes at least the sender of the geocast message and the target region of the message. It should also include a routing path between source node and target region. Otherwise, protocols either have to increase the size of the forwarding zone or fall back to simple flooding. An intermediate node forwards a message only if it belongs to the forwarding zone. In [21], the network is partitioned using the Voronoi diagram concept (to define forwarding zone) and each node forwards the packet to the neighbors whose Voronoi regions (as seen by the forwarding node) intersect with the geocast region. The idea is to forward to a neighbor only if it is progressively closer to the target region. In [22], the authors propose a geocasting algorithm detecting routing holes in underwater sensor networks. They use a similar approach to face routing and utilize x-y coordinates of the leaf nodes in the tree for detecting holes and face traversing. Although the authors show in simulations that the proposed protocol achieves handling of node mobility, there is no discussion about the mechanism that performs mobility handling. Interested reader are referred to [23] for a more comprehensive study of the related works.

In this paper, we consider the query dissemination problem in a tree-based data collection and dissemination network. Geographical approaches might either fail at dead-ends formed in random networks topologies or require the computation of planar subgraphs of the connectivity graph to tackle routing holes. On the other hand, tree-based approaches work well for random topologies with potential empty areas (*i.e.*, holes) in connected networks. Similar to GDSTR protocol [17], we also use a convex hull based approach for packet forwarding. However, the objective of GDSTR protocol is the node-to-node packet delivery realized by the use of multiple spanning trees rooted at different nodes for energy balancing. The main focus of [17] is on the choice of nodes serving as roots of spanning trees. There is not much attention to the construction and maintenance of hull trees. On the other hand, in this work we consider the case where the root of the tree is always the same node, which is a more resource rich device called data sink of the network. In this work, we provide the details of establishing convex hulls in the network and maintaining the routing tree and convex hull definitions in mobile sensor networks. We also compare different maintenance mechanisms (*i.e.*, global and local updating). By using convex hull based coverage areas and performing local updates, our approach provides support for sensor mobility.

In Section 5.2, we evaluate the performance of our geocasting approach (called GeoCHT), which utilizes coverage areas of sinks and sensors, by comparing it with GEAR protocol. The performance of different geocasting protocols is compared in [3] and GEAR shows the best performance over all the others. Therefore, we chose GEAR for our comparative performance evaluation. Simulation results show that GeoCHT outperforms GEAR in all metrics but query execution ratio in mobile sensor networks. Therefore, execution ratio is further evaluated together with communication overhead to investigate the performances of global and local updates in mobile sensor networks in Sections 5.4 and 5.5.

### 2.3. Multi-Sink Partitioning in WSNs

Multiple sinks (multi-sink) usage appears as a solution for large scale networks [24,25] since it provides some advantages such as energy efficiency, reliability and alleviation of the uneven energy depletion problem of a single-sink deployment [1]. However, deploying more sink nodes in a WSN brings another question: How to partition a sensor network among multiple sinks? The simplest way is for each sink to propagate a message to the whole network to form partitions. However, global flooding from each sink is redundant and costly. To reduce message redundancy and hence the flooding overhead, some flooding scoping techniques such as TTL (time-to-live) scoping or geographical scoping are used. TTL scoping defines a time-to-live for messages disseminated from sinks. In geographical scoping, a node only reforwards a flood message if the message came from the closest sink, where “closest” means shortest Euclidean distance. One of the geographical scoping methods proposed in [26] is Voronoi decomposition, where scoping decision is entirely distributed. This method describes Voronoi clusters to bound the propagation of messages from different sinks. Each node only rebroadcasts flood messages coming from closest sink, where “closest” depends on the underlying distance metric. With this approach, in the steady-state of a network (*i.e.*, only after each sink has originated one message) flooding overhead remains constant independently of the number of sinks [26].

The main focus of this paper is how to maintain the routing trees together with local coverage areas when we have mobile nodes in the sensor network. Our geocasting and mobility management approaches proposed in this paper can be combined with any of the multi-sink partitioning technique using any tree-formation metric to create routing trees rooted at the sinks. In our implementation we have used shortest path routing metric to create the trees, and Voronoi decomposition based on hop-count metric for multi-sink partitioning. The following give the details of our geocasting protocol based on local coverage areas maintained by global updates and describe the new supporting approach, local updates for handling sensor mobility.

## 3. Geocasting Based on Local Coverage Areas

In this section we briefly discuss the design of distributed coverage area construction that is proposed in our previous work [5,6]. Throughout this work, it is assumed that one or more sinks are deployed within the wireless sensor network and each sensor node is logically grouped with only one sink based on a given metric, e.g., shortest path. Basically, the routing strategy of the wireless sensor network determines which node reports to which sink. Before building the coverage areas, the network forms routing trees for each sink based on a given metric. In this work, we consider Voronoi decomposition (explained in Section 2.3) for partitioning of the network between sinks in which the underlying routing metric is the shortest path [4]. In the shortest path routing, a *child node* selects the neighbor that has the minimum hop count to a sink as the *parent node* (*i.e.*, the intermediate node) in order to get messages towards the sink. The construction of local coverage areas starts after the routing trees are created.

### 3.1. Establishing Local Coverage Areas

We assume that each node in the wireless sensor network has the ability to obtain an estimate of its position. Whenever a node publishes information, it is augmented with the current position of the node. An overview of the presented approach of establishing coverage areas is depicted in Figure 1. Nodes keep track of coordinates that are explicitly transmitted. Using the received coordinate information, a node creates its local coverage area. By the term *coverage area*, we understand the geographical area in which the sensor nodes are deployed. In this work, the coverage area of a node is represented by a convex hull [27] that envelopes the locations of the node itself and all its descendant nodes in the tree. The convex hull for a set of points is the minimal convex polygon that contains all the points; it is minimal because the convex hull will be contained in any convex polygon that contains the given points. The local coverage area of a node is the convex hull of the subtree rooted at this node (e.g., the subtree rooted at node x is covered by the red convex polygon and the subtree of node y is represented by the green convex polygon in Figure 1).

For the distributed construction of convex hulls along the routing tree, each node receives the convex hull information from all its child nodes and merges them together with the addition of the location of the node itself. This procedure constructs a new composite convex hull, which is forwarded further up the tree until it reaches to the sink. The convex hull of the sink covers all the sensor nodes served by this sink as shown in Figure 1 with the blue convex polygon having seven vertices.

Let us denote by **H_i_** the convex hull representing the (local) coverage area of sensor node or sink *i*. Let *p_i_* be the position of node *i* in the wireless sensor network. Initially, **H_i_** = {*p_i_*} contains the coordinate of the node *i* itself. However, during the update (*i.e.*, merging) process described below, the coordinate of the node itself might be removed from **H_i_**. After the update process, **H_i_** defines a convex hull of *n* vertexes over **C**, where **C** is a set of coordinates of *N* nodes (*i.e.*, the descendant nodes and the node *i* itself). **H_i_** covers all *N* nodes in **C**. The node *i* stores the list of vertices of the convex hull of **H_i_** in counterclockwise order for the computations in convex hull merging algorithm. Let |**H_i_**| and |**C**| denote the number of elements in the sets **H_i_** and **C**, respectively. Note that |**H_i_**| = *n*, |**C**| = *N*, and |**H_i_**| ≤ |**C**|. Apparently, storing a local coverage area represented by a convex hull uses only *𝒪*(*n*) storage instead of *𝒪*(*N*) storage.

In literature, many methods are described that transform a set of coordinates to a convex hull, e.g., [28]. Typically, these algorithms operate on a set of coordinates and produce a convex hull, but most of them do not consider addition of coordinates once the convex hull has been created. In fact, to construct convex hulls along the routing tree, we need an algorithm which implements a merge function *𝒞ℋ*(**H, C**) = **H**^′^, where **C** can be ***(i)*** a single coordinate, ***(ii)*** a coordinates set or ***(iii)*** a coordinates set representing a convex hull, with **H**^′^ ⊆ **C** ∪ **H**.

The simplest and the most efficient way to achieve merging of two convex hulls (*i.e.*, case ***(iii)***) is applying the rotating calipers based algorithm [29,30] locally in each parent node. . However, rotating calipers method cannot handle the merging of a convex hull with a single coordinate (*i.e.*, case ***(i)***) or a set of coordinates (*i.e.*, cases ***(ii)***). In our previous work, we present an algorithm that performs incremental construction of convex hulls, which covers for all the three cases discussed above. For incremental construction of a convex hull, we need to check if the new coordinate is inside the convex hull. This is done by checking whether the coordinate is always on the left side of each edge of the convex hull, where the coordinates of the convex hull are in counterclockwise order. The details of the algorithm can be found in [6,23].

With this “crude” coverage area, details such as holes in the wireless sensor network deployment become lost. Many geographical routing protocols (Section 2) need to take special precautions to ensure that messages are not stuck at holes in the deployment. However, the coverage areas are built based on connectivity information captured in routing trees; therefore, the holes are implicitly avoided.

### 3.2. Global Updating Mechanism for Building Trees and Local Convex Hulls

To construct and keep up-to-date routing trees and local convex hulls, a *global updating* mechanism is executed periodically with a frequency *f*. The value of frequency *f* is important to keep up-to-date routing structure. The effect of frequency *f* of global updating on the performance of our geocasting protocol is evaluated in Section 5.4. The periodic *global updates* consist of two phases: (i) *helloing from sinks* for tree building and multi-sink network partitioning, and (ii) *convex hull forwarding* to the parent nodes for completing the structure with local convex hulls of sensors and sinks.

In the first phase, each sink repeats broadcasting “hello” packets every *f* (*i.e.*, frequency) seconds to determine the paths to every node in the network and constructs a routing tree based on a given metric. In this work, we use the minimum hop count metric for tree construction. At the end of helloing process, each sensor node knows its hop count to each sink in the network. We use the definitions *parent node* and *child node* to indicate node positions in a routing tree. A child node selects a parent node as intermediate node in order to get messages towards a sink after helloing process. Minimum hop count metric results in the shortest path routing trees, which are constructed by having each node choose the neighbor with the minimum hop count to a sink as its parent. As a result, each node connects itself to the closest sink in terms of hop counts. Details and simulation results of different routing metrics (e.g., energy level) can be found in [23]. After routing tree construction, every node stores the logical address of its parent node in the tree, the sink it is connected to, and the hop count level from the sink (*i.e.*, HCL). Nodes do not need to keep track of their (possibly many) child nodes. The reason for this is to keep the minimal necessary information for routing in order to minimize storage of a sensor node.

In the second phase each node sends its parent a message called HELLO_TO_PARENT that includes the node’s convex hull. Periodically, the local convex hull is transmitted to the parent node. For distributed construction of convex hulls along routing tree, every parent node receiving a convex hull from its child node merges the received convex hull with the local convex hull of itself. This procedure constructs a new composite convex hull, which is forwarded further up in the tree until reaching to the sink. Optionally, the convex hull is reduced using some form of compressing before transmitting (in order to limit memory usage by our protocol and energy consumption by reducing the size of transmitted/received coordinate list) as explained in our previous work [6].

### 3.3. Maintenance: Removing Invalid Coordinates from Local Convex Hulls

Due to dynamics in network topology, the local convex hull stored in a node can contain coordinates that do no longer reflect the actual coverage area of the node. This might be the case when a node dies/fails or nodes are mobile. To keep the local convex hull up-to-date between two global updates, a timeout mechanism is applied to remove invalid coordinates from the local convex hull. Nodes store a timestamp for each individual coordinate in their local convex hull **H_i_**. The timestamp of a particular coordinate in **H_i_** is reset when node *i* receives a message (*i.e.*, HELLO_TO_PARENT) containing the coordinate. But when a coordinate has not been reinforced within the timeout interval, it is removed from the local convex hull **H_i_**. Node *i* sends its new convex hull to its parent node in its next HELLO_TO_PARENT message. When the parent hears about the changes, it will update its convex hull accordingly.

The *timeout interval* must not be shorter than the interval at which nodes produce and send their convex hulls in HELLO_TO_PARENT message to their parents, otherwise coordinates are removed from the local convex hulls before they are reinforced. If topology changes are frequent, the *timeout interval* should be short to ensure up-to-date coverage areas. Also, the local convex hull needs to be transmitted to parent nodes at least once per timeout interval.

To limit resource consumption, such as memory, energy, and bandwidth, of the proposed mechanism for coverage area reporting, compression of convex hulls (*i.e.*, approximation of the convex hull with a smaller coordinate set) is an attractive option. When reduction is only applied on copies of convex hulls forwarded to parent nodes, the timeout mechanism of coordinates remains functional without having to e.g., match coordinates to substituted coordinates. In any case, the parent node works with the compressed version. This implies that compressed local convex hulls need to be forwarded to parent nodes within the reinforcement period of coordinates to ensure that substituted coordinates are not removed due to timeout mechanism.

The timeout mechanism mainly repairs the affected convex hulls when nodes fail/die or move out the coverage area. The main functionality of this mechanism is removing old information. However, it can not totally repair the routing structure when there are moving nodes in the network. With this mechanism, a mobile node can be removed from its previous neighborhood, but it can not be added to its new neighborhood. In our previous work, for the addition of mobile nodes to their new neighborhood in the routing tree (*i.e.*, finding a new parent and updating convex hulls), the *global updating* mechanism described in Section 3.2 has to be executed periodically.

### 3.4. Geocasting Using Local Coverage Areas

With the above described algorithms, the sinks are informed of a “crude” description of their coverage area. Next, this information can be used to optimize handling of position dependent messages e.g., sinks can use the information whether a certain query is relevant for their coverage area. If not, the sink discards the query without inserting it in the WSN, which in the end saves energy and prolongs the lifetime of the wireless sensor network. In this section, the geographical routing of location dependent queries is discussed.

Queries are always forwarded from parent nodes to child nodes to get delivered to an area that is specified in the query. Let **R** = {*r*_0_, *r*_1_,…,*r_n_*} be the coordinate set describing the region of interest extracted from the query, **H_i_** the local coverage area of node *i* and *p_i_* the position of node *i*.

**Figure 2.**
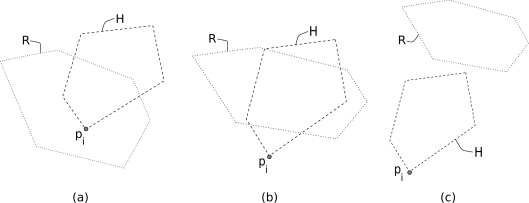
Routing decisions: (a) node *i* executes query if *p_i_* inside **R**, (b) node *i* forwards query to children if **H_i_** overlaps with **R**, and (c) node *i* halts forwarding query to children if **H_i_** and **R** are disjoint.

Upon receiving a query, a node analyses **R** and takes two decisions: (1) *execute decision* when the node is within the region of interest and (2) *forward decision* when the node has child nodes or further descendants in the region of interest. Both decisions use **R** as input together with *p_i_* and **H_i_**, respectively (Figure 2).

#### 1) Execute Decision:

It basically checks if the node that receives the query is inside the region of interest, *i.e.*, if point *p_i_* is inside the polygon **R**. The point-in-polygon problem is a well-known problem in computational geometry and many solutions and implementations have been proposed [31]. Complexity is reduced when the region of interest **R** is a *convex* polygon. To check if the coordinate *p_i_* is inside a convex polygon **R**, node *i* needs to check for every line segment the coordinate *p_i_* is left of the line segment. If so, the query needs to be executed (see Figure 2(a) and [6] for more details). In the simulations we assume that the target region is a circle having a radius *r_C_*. The node needs to check the distance between its coordinate *p_i_* and the coordinate of the center of the area of interest. If the distance is smaller than *r_C_*, the node should execute the query.

#### 2) Forwarding and Halting Decisions:

With the forwarding decision a node determines if there *might* be child nodes or descendant nodes further down the routing tree that are within the area of interest specified in the query. If there are, the node should forward the query to its child nodes, which in their turn decide if the query needs to be propagated. When the coverage area is represented by a convex hull, a node *i* cannot determine with certainty that there is indeed any node that is located in the subtree of node *i* and within the polygon **R**, because positions are lost for the nodes located within the convex hull. However, a node is able to decide with certainty that further in its part of the routing tree no node is present within the region **R**. When **R** is disjoint from the node’s convex hull **H_i_** in the later case, the node does not forward the query and it consequently does not spend energy on transmitting the query and also saves resources from its child nodes.

The forwarding decision is taken based upon **R** extracted from the query and the local coverage area **H_i_**. Checking if **R** overlaps with **H_i_** (*i.e.*, determining if two convex polygons intersect) can be easily performed in *𝒪*(log (*m* + *n*)) as described in [32], where *m* and *n* are the numbers of vertexes of the two polygons, respectively. We assume that the target region is a circle in our simulations. Checking if circle **R** overlaps with **H_i_** is done by “partially” checking the circle against edges [33], and against vertexes. If the area of interest and the local coverage area intersect, the node forwards the query (see Figure 2(b)). Otherwise, it halts the forwarding of the query (see Figure 2(c)).

## 4. Local Updates to Handle Mobility of Sensors for Geocasting

For handling changes in routing trees and local convex hulls due to mobility of nodes, the global updating is triggered periodically by sinks. One of the drawbacks of this approach is that a mobile node has to wait for the next global update for addition of itself to its new neighborhood, *i.e.*, selecting a new parent. Until next global updating, the mobile node will be disconnected from the network since it does not have a parent. Moreover, global updates periodically rebuild all routing trees and the local convex hulls of all nodes to ensure the routing structure is up-to-date. This approach updates even the tree branches which are not affected by the mobile nodes and consumes energy for unnecessary updates.

In this work, we introduce *local updates* initiated by the mobile sensors to update only the subtrees affected by mobility of nodes. In local updating, each mobile node decides its updating frequency based on its mobility and therefore does not have to wait for a global mechanism to select a new parent node. Local updating provides a continues process to keep track of changes in the network. With local updating mechanism, the removal of a mobile node from its old neighborhood and the addition of a mobile node to its new neighborhood are done in a timely manner. In addition, local updating mechanism is more efficient since local updates are limited in scope meaning that only the tree branches affected by mobility are updated. In the following, we give the details of the local updating mechanism.

**Figure 3.**
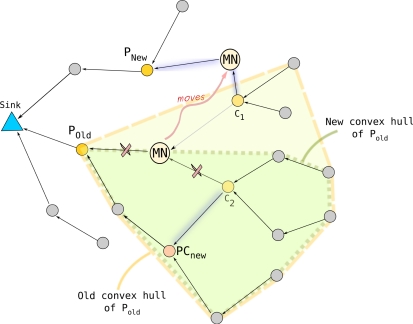
Effects of movement of MN on the routing tree and convex hulls.

When a mobile node ( MN) moves from one location to another, it may change its neighborhood, including its parent and child nodes. As result of mobility, the child nodes of the MN may need to re-associate themselves to another parent node and/or the MN may need to re-associate itself to a new parent node. Figure 3 shows an example of nodes and local convex hulls that are affected by the mobility of a node. MN and one of the child nodes (*i.e.*, C_2_) have to associate themselves with new parent nodes (*i.e.*, P*_New_* and PC*_new_*) after the movement of MN. Also, the convex hulls of new parent nodes (*i.e.*, P*_New_* and PC*_new_*), and old parent nodes (*i.e.*, P*_old_* and MN) change due to mobility. In the following we explain how to detect these changes by the neighbors of MN and how to update accordingly the tree and the convex hulls of the affected nodes.

**Figure 4.**
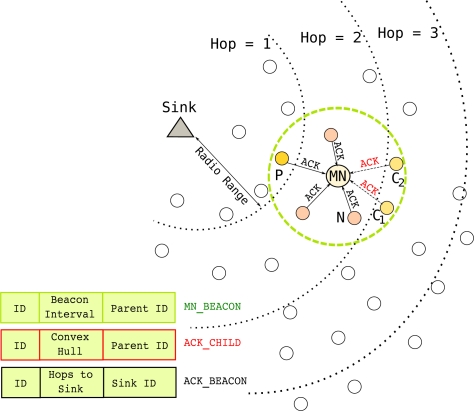
Beaconing to handle mobility of MN in the routing tree.

As shown in Figure 4, the neighbor list of a MN consists of the child nodes (*i.e.*, Node C_1_ and C_2_) and a parent node (*i.e.*, Node P) of MN and the other close-by nodes (e.g., Node N). To inform neighbors about its movement, MN periodically broadcasts *beacon* packets called MN_BEACON. The frequency of MN beaconing depends on the transmission range, speed of MN, and a parameter *k* (*i.e.*, *beaconing parameter*) reflecting the characteristics of the network. The following equation shows how the frequency of MN beacon packets is calculated:

(1) $$\text{MN}\_\text{BEACON}\_\text{INTERVAL}\;=\;\frac{R_{\mathrm{TX}}}{V_{\mathrm{MN}}}\times k$$

where 0 < *k* ≤ 1, *V_MN_* and *R_TX_* denote the maximum mobile node speed (m/s) and the maximum radio range (m), and $\frac{R_{\mathrm{TX}}}{V_{\mathrm{MN}}}$ is the time to leave the transmission range. The parameter *k* helps to determine how often a MN_BEACON packet should be sent to achieve prompt updates. It can be designated based on the network characteristics such as node density. The effect of the parameter *k* on the performance of the local updating is evaluated with simulations in Section 5.5. It is important to note that MN_BEACON_INTERVAL is the local update rate since it is determined by the speed of MN. Each mobile node in the network has its own beaconing (updating) interval. Figure 4 shows the content of a MN beacon packet. An MN_BEACON packet contains the ID of MN, frequency of the beaconing, and parent ID of MN.

**Figure 5.**
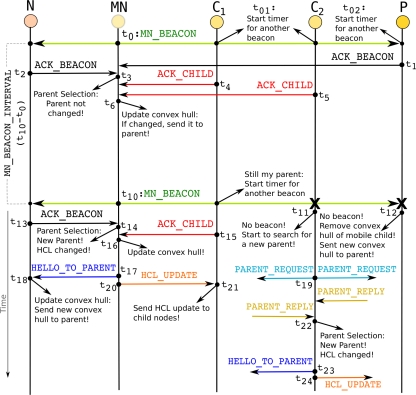
Messaging during local updates.

Messaging during the local updates is illustrated in Figure 5. In the figure, MN broadcasts a MN_BEACON packet at time t_0_. The receivers of a beacon packet first check who sent the beacon. The receiver node can be (i) the parent node P of MN, or (ii) a regular neighboring node N of MN, or (iii) a child node C of MN. Every node that receives a MN_BEACON packet sends an acknowledgement (*i.e.*, ACK) message back to MN initiating the beaconing as shown in Figure 4. However, the content of the acknowledgement message is determined by the relationship between MN and the receiver node:

- When a regular neighboring node N or the parent node P of MN receives a MN_BEACON packet, the receiver node sends an ACK_BEACON packet back to MN. Figure 5 shows that the parent node P and the neighbor node N send ACK_BEACON packets back to MN at times t_1_ and t_2_, respectively. An ACK_BEACON packet includes the ID of the node, the sink ID which this node is connected to, and the number of hops to the sink as shown in Figure 4.
- When a child node of MN receives a beacon from MN, the child node sends an ACK_CHILD packet back to MN. Figure 5 shows that the child nodes C_1_ and C_2_ send ACK_CHILD packets back to MN at times t_4_ and t_5_, respectively. An ACK_CHILD message includes the convex hull (*i.e.*, coordinates of convex hull) of the child node as shown in Figure 4.

It is important to point out that when the parent node or a child node of MN receives a beacon, it starts to wait for another beacon from MN (at times t_01_ and t_02_ in Figure 5). After the beacon interval passes, MN sends another MN_BEACON packet at time t_10_ as shown in Figure 5. If the child node of MN does not get another beacon (since MN moves out of communication range of the child node) in the time period (beacon interval) specified in the MN_BEACON packet, it starts to *search for another parent* at time t_11_ (details in Section 4.5). If the parent node of MN does not receive another beacon within the beacon interval (since MN moves out of communication range of the parent node), it *removes the convex hull of* *MN* *from its local convex hull* at time t_12_ (details in Section 4.4).

**Figure 6.**
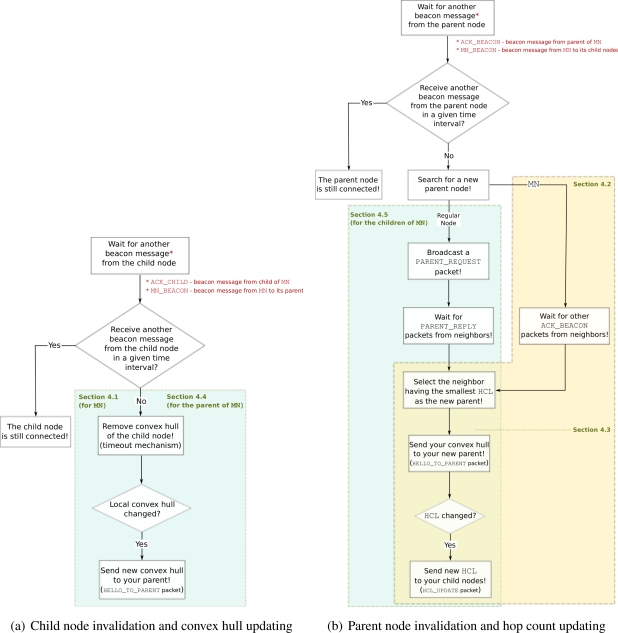
Fundamental operations of local updating mechanism.

After waiting for ACK packets from its neighbors, MN executes the following actions: ***(i)*** If MN does not get an ACK from a child node, it updates its convex hull and propagates the changes up on the tree (child node invalidation, see Figure 6(a)), and ***(ii)*** If MN does not get an ACK from its previous parent node, it selects a new parent and propagates the convex hull up and the new hop count level down the tree after new parent selection (parent node invalidation, see Figure 6(b)).

From Section 4.1 to Section 4.5, we mainly discuss two fundamental operations of local updating mechanism: (i) Child node invalidation and convex hull updating, and (ii) Parent node invalidation and hop count updating, which are needed to keep routing structure up-to-date. Figure 6(a) shows the flow chart of “*child node invalidation and convex hull updating*” which are explained in Section 4.1 for MN and in Section 4.4 for the parent of MN. The flow chart of “*parent node invalidation and hop count updating*” is given in Figure 6(b) as explained in Sections 4.2 and 4.3 for MN and in Section 4.5 for the child nodes of MN.

### 4.1. Updating the Convex Hull of MN and Propagating Changes

If a child node is not in the communication range of MN anymore, MN does not receive ACK packets from this child node. Therefore, the convex hull of this child node should be removed from the convex hull of MN. For a MN to maintain its convex hull, it calculates its new convex hull after it receives new ACK_CHILD messages from its child nodes which are still connected to MN (at times t_6_ and t_16_ in Figure 5). There are two options for updating MN’s convex hull: (i) MN can use a timeout mechanism by utilizing ACK_CHILD packets; if there is no ACK_CHILD for a given interval from a specific child, MN removes the convex hull of this child (similar to timeout mechanism explained in Section 3.3), or (ii) it can wait for a predefined time interval and then calculate its convex hull from the received ACK_CHILD packets. If MN’s convex hull is changed [34], MN sends a convex hull update message (*i.e.*, HELLO_TO_PARENT) to its parent to inform the parent about its new convex hull, as shown in Figure 5 at time t_17_. Any node receiving a convex hull update message recalculates its convex hull and compares this new convex hull with the old one. If the node’s convex hull is changed, then it sends an update message (at time t_18_ in the figure) to its parent. This forwarding of update messages continues until changes reach to the root of the tree, the sink node.

### 4.2. New Parent Node Selection of MN

If MN receives an ACK_BEACON packet from its parent, MN concludes that it is still in the communication range of its parent. Although MN is still in the communication range of the current parent node, it may change its *hop count level* (HCL) due to its mobility, e.g., comes closer to the sink. However, as long as the current parent of MN is in the communication range, MN keeps the same parent node, although there exist other parent candidates having shorter distances to the sink. With this approach, we avoid sending of extra HCL update messages (*i.e.*, HCL_UPDATE) to the child nodes of MN, which are needed if MN connects to a parent with a different HCL. Also, MN still remains connected to the tree.

If MN does not receive an ACK_BEACON packet from its parent, MN concludes that it is not in the communication range of its parent anymore. Therefore, MN checks the HCLs (*i.e.*, hops to sink entries in ACK packets) of ACK_BEACON packets sent by other neighbor nodes. When MN receives an ACK_BEACON, it records the sender node as a candidate for the parent node. After the predefined time interval expires, MN checks its candidate parents list. Since its current parent is not in the list, it chooses the node with the smallest HCL from the candidate parents list as the new parent node. In Figure 5 at time t_14_, MN chooses node N as the new parent.

### 4.3. Convex Hull and HCL Propagation After MN Changes Its Parent

If the hop count level (HCL) of MN changes after the new parent selection, MN sends its new HCL (in HCL_UPDATE message at time t_20_ in Figure 5) to its current child nodes. The child nodes also update their HCLs to the sink. This HCL update message is propagated (at time t_21_ in the figure) until it reaches to the leaf nodes of the branch. Also, MN sends a HELLO_TO_PARENT message including it convex hull to its new parent. The parent node receiving a HELLO_TO_PARENT message recalculates its convex hull. The convex hull changes are propagated up the tree until the changes reach to the sink.

After receiving the first beacon packets from MN, child nodes and the parent node of MN start to wait for another MN beacon packet for a beacon interval. In the following, we explain the actions executed by the parent and child nodes of MN when there is no other beacon from MN.

### 4.4. Removing MN’s Convex Hull from Its Previous Parent Node

If the parent node of a MN does not receive another beacon packet from its mobile child after the beacon interval defined in the previous MN_BEACON packet, it concludes that it is not in the communication range of its mobile child anymore. Therefore, the convex hull of the mobile child should be removed from the convex hull of the previous parent of MN as illustrated in Figure 5 at time t_12_. The timeout mechanism for removing coordinates from local coverage areas is executed here to remove MN’s convex hull from the convex hull of its previous parent. It is important to point out that the timeout interval should not be shorter than the beaconing interval (*i.e.*, MN_BEACON_INTERVAL) to ensure that MN’s convex hull is not removed before it is reinforced. If the aggregated local convex hull of the previous parent of MN is changed after the removal of MN’s convex hull, the previous parent of MN also sends its new convex hull to its own parent node.

### 4.5. Parent Invalidation for Child Nodes of MN

If a child node of MN does not receive another beacon packet from its mobile parent after the beacon interval defined in the previous MN_BEACON packet, it concludes that it is not in the communication range of its parent anymore. Therefore, it searches for another parent. For this purpose, it broadcasts a PARENT_REQUEST message. The child node C_2_ does not receive another beacon from MN and at time t_19_ it starts to search for another parent by sending a PARENT_REQUEST message in Figure 5. All nodes receiving a PARENT_REQUEST message reply a PARENT_REPLY message back to the initiator of the parent request message. After the node receives PARENT_REPLY messages, it chooses the node which has the smallest HCL to the sink (at time t_22_ in the figure). Then, the child node sends a HELLO_TO_PARENT message to its new parent to inform about its convex hull (at time t_23_ in the figure). The new parent receiving a HELLO_TO_PARENT combines its convex hull with its new child node’s convex hull and propagates its new convex hull to upper nodes in the tree if the aggregated convex hull is changed. After the new parent selection, if the current HCL of the child node is different than its previous HCL, it also propagates its new HCL to its child nodes (at time t_24_ in the figure) and the whole branch down in the tree.

Several factors contribute to the fact that wireless sensor networks often do not work as expected when deployed in a real-world setting. Some of them are environmental influences which may lead to non-deterministic behavior of radio transmission, and therefore, packet loss. In case of packet loss, our local updating mechanism can still function. If a beacon packet (*i.e.*, MN_BEACON, ACK_CHILD) from a child node is lost, the convex hull of this child node can be removed from the parent temporarily. However, in the next beaconing the child node is reinforced in the parent node. If a beacon packet (*i.e.*, MN_BEACON, ACK_BEACON) from a parent node is lost, the child node can connect itself to another node from its neighborhood. Therefore, packet loss does not have much influence on basic functions (*i.e.*, query and data routing) of the network although there might be temporary child-parent node disconnections. The performance evaluation metrics, which are used in the next section, are independent of imperfect conditions of wireless transmission such as packet losses.

## 5. Performance Evaluations

In order to evaluate the performance of global and local updates described above, we used the open source network simulator NS-2 [35] version 2.33 as it is widely used for research in wireless sensor networks. We have added a new routing agent (*i.e.*, GeoCHT) into NS-2 over the currently implemented network stack.

In the following, we provide first a description of the metrics for evaluating the performance of the proposed mobility handling methods and for evaluating the routing accuracy and networking performance of the convex hull based geocasting. Next, we compare our convex hull based geocasting (*i.e.*, GeoCHT) with another geocasting protocol, GEAR, in terms of routing accuracy and networking performance. After that, we present the scenarios characteristics for evaluation of the performance of the proposed mobility handling method. Finally, we analyze the obtained simulation results.

### 5.1. Evaluation Metrics

The proposed mobility handling method for our geographical routing protocol is evaluated in terms of routing accuracy *i.e.*, how well the proposed mechanism delivers messages to the region of interest defined in a query and in terms of communication overhead. The performance of two methods (*i.e.*, global updates and local updates) for handling mobility in geocasting has been evaluated by varying the number of nodes, number of mobile nodes and speed of the mobile nodes with the following scenarios:

- *Scenario 1*—The aim of this scenario is to see the scalability of the updating methods. For this purpose, we vary the number of nodes from 60 to 360, where 10% of the nodes are moving with a speed of around 10 *m/s*. We keep the mobility rate and speed fixed while increasing the network size to evaluate the performance of updating mechanism in terms of routing accuracy and energy efficiency for networks of different sizes.
- *Scenario 2*—This scenario is evaluated to see how well the updating methods can handle the dynamics of the network. If more nodes are moving in the network, more parts of the routing structure are affected by the mobility. We vary the number of mobile nodes from 10 to 50 in a network having 200 nodes. The speed of the mobile nodes is around 10 *m/s* reflecting moderately moving entities.
- *Scenario 3*—Higher speed of nodes results in more frequent changes in the routing structure. To understand how accurate the updating methods (for keeping the routing structure up-to-date during the network lifetime) are, in this scenario we vary the speed of mobile nodes from 1 to 17 *m/s* in a network having 200 nodes with 20 mobile nodes. The speed of mobile nodes is varied from slowly moving entities such as firefighters (with a speed of 1–2 *m/s*) to highly mobile entities such as UAVs (with a speed of 15–17 *m/s*) in a firefighting scenario.

In these multi-sink WSN scenarios, we have measured two metrics to evaluate the mobility handling mechanism for routing of queries towards a specific region:

- *Execution ratio (ER)*—The ratio of nodes that are within the region of interest and execute the query with the total number of nodes within the region of interest. This metric measures how well the routing is able to deliver the query to the region of interest. It is defined to evaluate routing accuracy of queries in mobile WSN.
- *Communication Overhead (CO)*—The total number of packets that are sent to construct and maintain the structure. It includes hello packets sent from sinks, beacon packets sent from mobile nodes and convex hull definition update messages. If more messages need to be transmitted to keep the geocast structure up-to-date, the energy expenditure in the WSN is likely to increase. We measure this effect with overall network load.

Two other metrics are used to evaluate performance of geocasting in terms of routing accuracy and compare it with other known geocasting protocols:

- *False execution ratio (FER)*—The ratio of nodes that are outside the region of interest and execute the query with the total number of nodes outside the region of interest. Energy is wasted when queries are executed outside the region of interest. The false execution ratio measures this effect.
- *False injection ratio (FIR)*—The ratio of data sinks that inject the query while none of the nodes in its partition executes the query with the total number of data sinks. Irrelevant query lead to higher energy expenditure in the WSN partition when injected. We measure this effect with the false injection ratio.

We define two final metrics to evaluate networking performance of geocasting and compare it with other known geocasting protocols:

- *Average query delivery delay (AQDD)*—The total time elapsed between the query generation by a sink and its reception by a sensor node inside the target region, averaged over all sink-target node pairs.
- *Network load (NL)*—The total number of query packets that are sent from sinks and sensors until reaching to target region.

False injection ratio (FIR), False execution ratio (FER), Average query delivery delay (AQDD), Network load (NL) are used to evaluate the performance of convex hull based geocasting. However, since our aim is to evaluate the performance of discussed mobility handling techniques in a tree-based network, we are mostly interested in ER and CO which are directly related with mobility handling. The other metrics, which are not much affected by mobility, were extensively evaluated for a wide range of scenarios in our previous paper [6]. The effects of location estimate errors on geocasting can also be found in [6]. Here, to see the effectiveness of the geocasting based on convex hulls (called GeoCHT at the remaining of the paper), we provide a brief comparison of convex hull based geocasting with GEAR protocol in the next section. After that, we discuss the performance of mobility handling techniques, global and local updates, used by our protocol.

### 5.2. Efficiency of Convex Hull Based Geocasting

**Figure 7.**
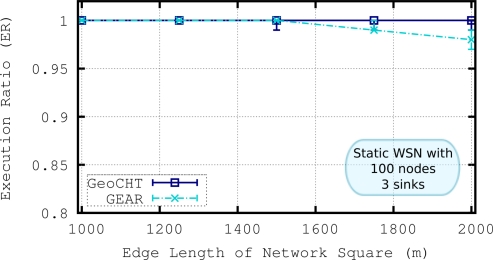
Execution Ratio of GeoCHT and GEAR in a static WSN.

The performance of the GeoCHT and GEAR is evaluated and compared in both static and mobile scenarios in our previous work [6]. The details of the GEAR protocol can be found in Section 2.2. In this section, we briefly summarize the results of this comparison to show the efficiency of GeoCHT. The simulation results show that GeoCHT performs quite well in static scenarios. AQDD and NL of GeoCHT are much lower than GEAR while GeoCHT has higher ER and lower FIR in most of the cases. Figure 7 shows the comparison of ERs of GeoCHT and GEAR in a static WSN with varying density. Note that an execution ratio of 1 means that every node inside the target region has received and executed the corresponding query packet. Since we focus on the mobile WSNs in this work, we present the comparison of GeoCHT and GEAR in a mobile scenario in the following discussion.

To show the effectiveness of the convex hull based geocasting (*i.e.*, GeoCHT), this set of simulations are perfomed in a 2,500 × 2,500 *m*^2^ deployment area with 200 sensor nodes and 6 sinks. We evaluate the effects of different number of mobile nodes in the network, namely 1% to 20% of the network nodes are moving. The average speed of the mobile nodes is 5 m/s. Global updating is used in this set of simulations to construct geocast structure. The queries are sent from sinks at a random time after the geocast structure is updated by the global updating mechanism.

**Figure 8.**
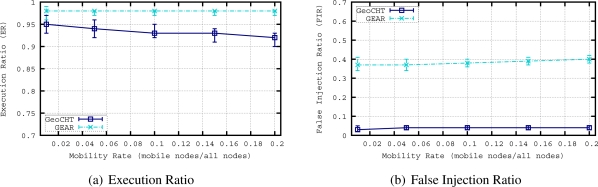
Routing accuracy performance with varying mobility rate.

Figure 8 shows the routing accuracy performance of the protocols. Simulations show that the False Execution Ratio of queries in mobile networks is zero for both GEAR and our protocol GeoCHT, therefore not shown in the graphs. The reason of this FER result is that when a mobile node, which was in the target region before starting to move, receives a query, it first checks its current position and if it is not inside the area of interest anymore, it does not execute the query. In Figure 8(a), we see the ER results of GEAR and GeoCHT. GEAR slightly outperforms GeoCHT when we increase the mobility rate (*i.e.*, number of mobile nodes). This is mainly due to the fact that we used tree-based shortest path routing to connect nodes to sinks in GeoCHT simulations. In mobile sensor networks, tree-based approaches require frequent reconfigurations. This result has motivated us to focus on mobility handling in convex hull based geocasting in this paper. The following sections evaluate the performance of two methods, *i.e.*, global and local updating to achieve efficient and on time reconfiguration of the geocast structure. Figure 8(b) presents the FIR results of GEAR and GeoCHT. The FIR of GEAR is higher than the FIR of GeoCHT in this mobile scenario. When we increase the mobility rate in the network, the FIR of GEAR slightly increases. GeoCHT also has a slight increase in FIR when the mobility rate is increased. This means that although convex hulls of sensors encapsulated in the convex hull of a sink are affected by mobile nodes, the coverage areas of sinks are not much affected by the mobile nodes. Only the movement of the nodes which are closer to the boundary of the convex hull of a sink may change the coverage area of this sink if they move out of the current convex hull of the sink.

**Figure 9.**
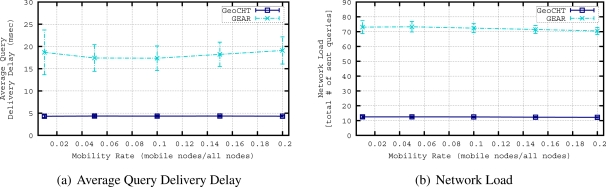
Networking performance with varying mobility rate.

Figure 9 shows the networking performance of the protocols. In Figure 9(a), we show the query delivery delays of both protocols. The delays are not affected much by the increasing number of mobile nodes in the network. The delay in GeoCHT is smaller than the delay of GEAR because GeoCHT discards the queries of sinks which are very far from the area of interest. Only the sinks that are close to the area of interest inject their queries; however, in GEAR, even if a sink is very far from the target region, it still injects its query so the average query delivery delay of GEAR is getting higher. Another reason of this difference in delays of GEAR and GeoCHT is their different forwarding mechanisms. GEAR uses unicasting to reach next hop but GeoCHT uses a special restricted broadcasting (*i.e.*, multicasting) which is quicker in the delivery of the message to the next node. The network load of GEAR and GeoCHT are shown in Figure 9(b). The network load of GEAR is higher than the network load of GeoCHT in this mobile scenario. The network load of GEAR is slightly decreasing when we increase the number of mobile nodes since for a node, the probability of changing its neighborhood is getting higher so the query packet can get stuck at a node which does not have a neighbor that is closer to the area of interest anymore. Therefore, the number of sent query packets decreases in GEAR.

As a results of this comparison, we can conclude that geocasting based on convex hulls requires less messages to be injected from sinks to deliver queries to a target area. Also, the latency for geocasting based on convex hulls is lower. These results point out that our protocol achieves a more efficient use of resources than pure greedy forwarding towards the target region and restricted flooding inside the target region based geocasting approaches (*i.e.*, GEAR). On the other hand, convex hull based geocasting needs a special mechanism to update the geocast structure in case of mobility of sensors to improve ER.

### 5.3. Scenario Characteristics for Mobility Handling Simulations

**Figure 10.**
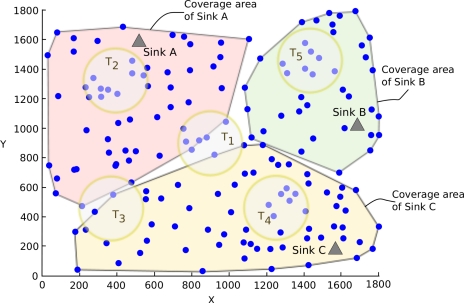
An example topology with coverage areas of sinks and target regions.

In the simulations, the sensor nodes are uniformly deployed in a rectangular deployment area of 1,800 × 1,800 *m*^2^ where we have 3 sinks. Every sensor node has a transmission range of 250 *m*. We use Random Waypoint movement model to simulate sensor node mobility. The speed of the mobile nodes varies from low mobility (*i.e.*, 4–5 km/h for walking humans) to high mobility (e.g., 40–60 km/h for UAVs [36]) scenarios. A simulation run lasts 100 simulation seconds. For a given simulation run, results are averaged over all the queries sent during the simulation. The averaged values are calculated over 10 different deployments with a fixed number of sensor nodes. For each deployment, we randomly select 5 different center points for disjoint target regions which are circular areas. Assuming to divide the deployment area into 4 sub-rectangles from the center, one of the target region is at the middle of the network and the other four are in one of the sub-rectangles. Figure 10 illustrates an example simulation topology with convex hulls of sinks and target regions. The target regions are created in a way that they cover all the directions in the deployment area. The simulation parameters are given in Table 1. Although IEEE 802.15.4 is a standard developed to meet the needs for low-power and low-cost wireless communication, we prefer to use the IEEE 802.11 standard in our simulations to be comparable with previous works. A comparative performance study of IEEE 802.15.4 and IEEE 802.11 can be found in [37]. Another reason of using IEEE 802.11 in the simulations is that it achieves a higher packet delivery ratio since it uses a handshaking mechanism before sending packets. IEEE 802.11 is chosen to minimize the effect of MAC layer on the performance of our routing protocol.

We compare the performance of our geocasting protocol when we have only global updates started by sinks periodically and local updates triggered by mobile sensors. Global updates are sent from sinks with a frequency varying from 3 to 25 seconds. In simulations with only global updates, the queries are sent 100 *ms* before the next global update starts. In simulations with construction of geocast structure followed by only local updates, the queries are sent every 10 seconds. As described in Section 4, mobile nodes send beacon packets periodically having a frequency based on the speed of the node and the mobile node beacon parameter, which varies.

### 5.4. Evaluation of Frequency of Global Updates

To estimate the most efficient global update frequency, we test different frequency values (*i.e.*, *f*) varying from 3 to 25 *s*. In this set of simulations, sinks perform periodically global updates to update the routing tree and local convex hulls. There is no local update during the simulations, *i.e.*, mobile nodes do not sent local update messages to their neighbors. Higher frequency of global updates means that the routing trees of sinks will be updated more frequently resulting in new geocast structure most of the time. The queries are sent just before the next global update starts to see the worst case behavior of different update frequency values.

**Figure 11.**
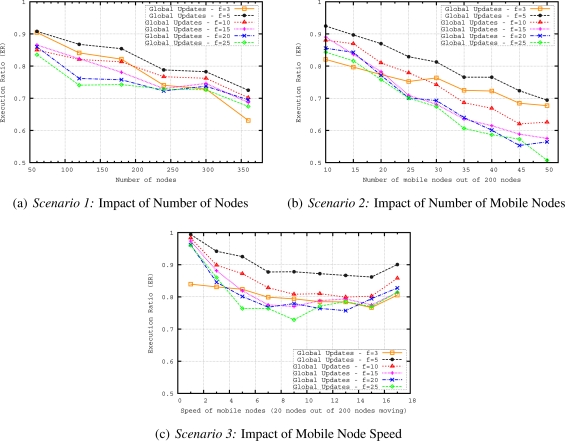
Execution Ratio for different frequencies of global updates.

In Figure 11(a), we evaluate execution ratios (ERs) of different *f* values when we increase the number of nodes and the number of mobile nodes in the network. We observe that increase in number of nodes and number of mobile nodes in the network results in decreases of ERs for different global update frequencies. The best ER is obtained when *f* = 5. Although global updating every 3 seconds refreshes the tree structure more frequently, the drop in the ER of *f* = 3 is very large when we increase the number of nodes. This is mainly due to the overlapping of global updates. Overlapping means that before finishing one global updating, the next one starts. The overlapping time gets longer when we have more nodes in the network. Therefore, for bigger network sizes 3 seconds is not enough to finish one global update. For instance, for a network having 360 nodes, it takes around 3.75 seconds to finish one global update. For other *f* values bigger than 5, ER is worse than ER of *f* = 5 for the given network sizes. It is important to point out that for networks bigger that 360 nodes, 5 seconds may also not be enough to finish one global update. However, it is the best frequency for the range of network sizes we consider here.

In Figure 11(b), which reflects the results of *Scenario 2*, we plot the dependency between ER and the number of mobile nodes. While in *Scenario 1* (Figure 11(a)), the ratio of mobile nodes to the number of nodes in the network is kept constant, here we vary the number of mobile nodes in the network and keep the total number of nodes fixed. ER of *f* = 5 is also the best in this scenario. In general, when we have higher mobility (*i.e.*, more mobile nodes) in the network, the ER decreases for all values of *f*.

Finally, in Figure 11(c), we explore the influence of speed of mobile nodes on ERs of different update frequencies. The frequency *f* = 5 again achieves the best performance. However, different than the other scenarios, ERs first slightly decrease when we increase the speed of mobile node up to 9 *m/s*, then ERs stay more or less stable or slightly increase for the speeds higher than 9 *m/s* in this scenario. This behavior is due to the adaptation of timeout mechanism according to speed of mobile nodes. For higher speeds, timeout interval is shorter; hence, up-to-date convex hull descriptions can be obtained quicker. Since the geocast structure is updated more frequently for higher speeds by the timeout mechanism, ER results are the same or better for higher speeds.

Maintaining up-to-date convex hull information by exchanging control packets incurs control overhead. Possessing the most up-to-date tree structure provides better chance to build a more efficient query dissemination solution. However, there is a trade-off with the update interval: if trees routed at sinks are updated too often, the benefit of having current state information diminishes and most of the energy will be consumed exchanging control packets (*i.e.*, hello, convex hull update messages). In what follows, we investigate the communication overhead of different update frequencies in the three scenarios.

**Figure 12.**
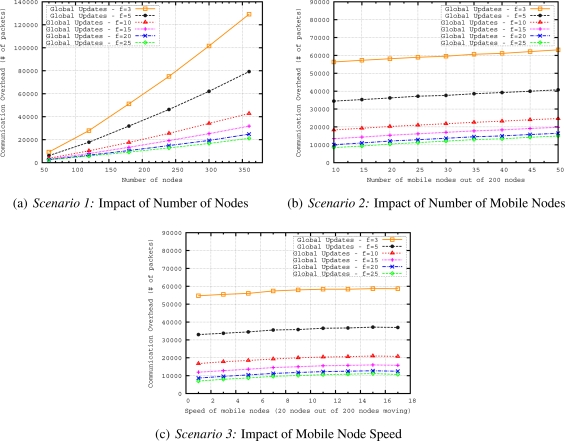
Communication Overhead for different frequencies of global updates.

Figure 12(a) presents the results in terms of communication overhead (CO) when we vary the number of nodes. CO values of global updating with *f* = 10, 15, and 20 are very close to each other. On the other hand, when we increase frequency to 5 and 3, the increase in the communication overhead is very large with increasing number of nodes. The trend in Figure 12(a) is steeper when we increase the number of nodes; on the other hand, in Figure 12(b) and Figure 12(c), the graphs increase gradually. In Figure 12(b), we observe that COs of all frequencies slightly increase. The reason for this behavior is that when we increase the number of mobile nodes, more convex hull update messages are generated due to higher mobility rate. Figure 12(c) shows the CO results with increasing mobility speed. The figure has a very similar shape with the varying number of mobile nodes graph in Figure 12(b). Again, due to adaptation of timeout interval according to the speed of MN, higher speed results in more convex hull update messages in the network.

### 5.5. Evaluation of Accuracy of Local Updates

In this set of simulations, only one global update (*i.e.*, sinks helloing) is performed at the beginning of the simulation to build a full geocast structure (*i.e.*, the routing trees and convex hulls) in the network. Then, only local updates from mobile nodes are sent in their neighborhoods to keep geocast structure up-to-date during the simulation. Performance of local updating with different *k* values are compared. The parameter *k* affects the frequency of local updates performed by MNs. A small *k* results in triggering local updates more frequently. Assuming a mobility speed of 10 *m/s* and 250 *m* transmission range, when we have *k* = 0.10, MN sends a beacon packet for every 25 *m* displacement.

**Figure 13.**
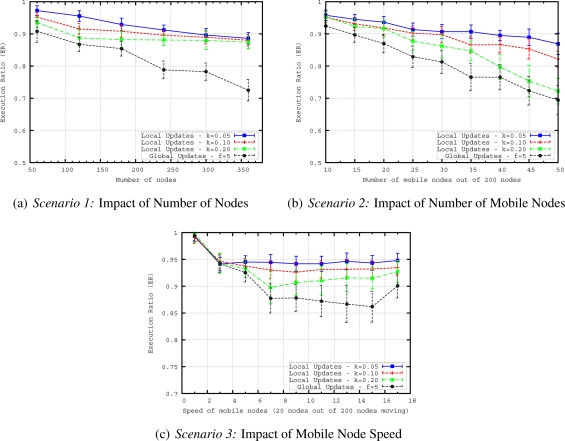
Comparison of local and global updates in terms of Execution Ratio.

**Figure 14.**
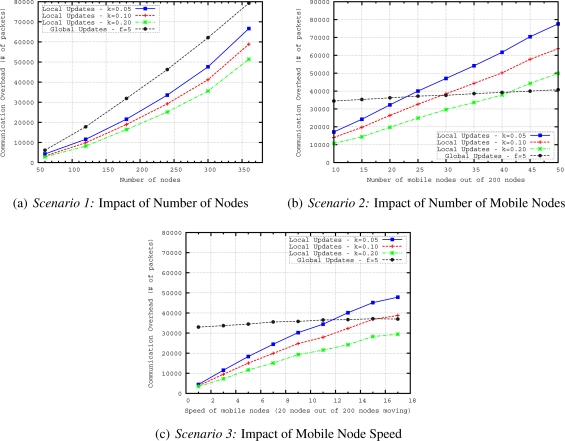
Comparison of local and global updates in terms of Communication Overhead.

In Figure 13(a) we compare the local updates with different MN beacon parameter, *k*, values and global updating with a frequency of 5 seconds which achieves the best performance as shown in the previous set of simulations. It is observed that ER of all local updates with different *k* values is better than the best global update. When we increase the number of nodes in the network by keeping the deployment area fixed, the density of the network also increases. This increase results in shorter distances between parent and child nodes. Hence, as observed in the figure, for bigger number of nodes, the ER values are very close to each other for different values of *k* because longer MN beacon intervals still achieve on-time updating of geocast structure in dense networks. With this observation, we can redefine *k* as *density factor* since it is determined by the density of the network. In Figure 14(a), we compare the communication overhead (CO) of local and global updating. When sensor nodes are more densely deployed in the sensor field, the CO increases for both global and local updating. However, the CO of local updating with different *k* values is always lower than that of global updating. This is an expected results since local updates only reshapes the branches affected by mobile nodes so the messaging is reduced in the local updating.

Figure 13(b) plots the ERs of local and global updating when we increase the number of mobile nodes in the network. Local updating with *k* = 0.05 gives the best results of ER. While ERs of local updating with *k* = 0.05 are very close to local updating with *k* = 0.10 and *k* = 0.20 for networks having less than 35 mobile nodes, this behavior changes for networks having more mobile nodes. Results show that when we have more mobile nodes, more frequent updates are needed to reflect the actual geocast structure. For any *k* value of local updating, global updating gives worse ER results than local updating. Figure 14(b) presents the results in terms of communication overhead when we vary the number of mobile nodes. As expected if we have more number of mobile nodes, the messaging and communication overhead increases for local updating. On the other hand, CO slightly increases for global updating when we increase the number of mobile nodes since communication overhead of global updating mainly depends on the number of nodes in the network and some convex hull update messages due to timeout mechanism. However, as observed from Figures 13(b) and 14(b), it is possible to determine a *k* value for local updating which results in less CO and still performs better than global updating in terms of ER. If we compare the COs of local and global updating, for example for *k* = 0.20, when we have 40 mobile nodes in the network, COs of local and global updates are more or less the same. This number is getting smaller for smaller *k* values (*i.e.*, COs of local and global updates are similar when we have 25 nodes for *k* = 0.05 and 30 nodes for *k* = 0.10) of local updating because smaller *k* values create more beacon and update messages in the network.

In Figure 13(c), we evaluate ERs of local and global updating when we increase mobility speed. ERs of local updating with different *k* values first slightly decrease when we increase the speed of mobile nodes up to 9 *m/s*, then ERs slightly increase or stay unchanged for the speeds higher than 9 *m/s*. For higher speeds of mobile nodes, MN_BEACON_INTERVAL gets shorter and mobile nodes become the leaf nodes of the trees more quickly since they send leave messages to their child nodes quickly after they start to move. In Figure 14(c), we explore the influence of speed of mobile nodes on COs of local and global updates. When we increase the speed of mobile nodes, COs of local updates with different *k* values increase. This is due to the fact that higher speed means shorter MN_BEACON_INTERVAL; thus, more MN beacon and convex hull update messages for local updating mechanism. On the other hand, CO of global updating slightly increase with increasing speed. CO of local updating with *k* = 0.20 is always smaller than CO of global updating; however, when we assign *k* = 0.10, the COs of local and global updating are very similar for the speed of 15 *m/s* (*i.e.*, around 55 *km/h*).

### 5.6. Discussion of Simulation Results

As we observed from the simulation results, local updates perform very well in terms of ER; on the other hand, local updating is much more energy efficient than global updating in networks having any number of nodes, but, small number of mobile nodes (*i.e.*, around 10–15% of the number of nodes) moving with a speed varying from low to high (*i.e.*, around 1–20 *m/s*). This specification is very much valid for mostly static sensor network scenarios in which most of the sensors are static and some sensors are attached to people or vehicles such as firefighters or firetrucks, unmanned aerial vehicles (UAVs) moving at low or medium velocities in an Emergency Response Application.

It is possible to apply some improvements to local updating mechanism if we assume that the mobile nodes are aware of their direction of movement. Mobile nodes can easily determine their movement direction, if they are aware of the position of the sink node. Some of the acknowledgement packets can be eliminated when a mobile node knows whether it moves towards the sink or in the opposite direction. If mobile node moves towards the sink, there is no need to have acknowledgements from the neighbor nodes in the same hop count level. Since the number of acknowledgement packets increases as the network size gets bigger (*i.e.*, the number of neighbors increases), reducing unnecessary acknowledgement packets will have a positive impact on the overall communication overhead.

## 6. Conclusions

We presented a protocol for routing of queries in a region of interest. The protocol relies on a structure that consists of routing trees and convex hulls of tree branches and the full trees, which represent the respective coverage areas. This structure is used for an efficient routing of queries. This paper focuses on handling mobility where several sensors move in the sensor field. The mobility imposes high needs in the updating of the geocast structure. We proposed a local update mechanism, where updates are triggered by moving sensor nodes and are kept constrained only to the parts of the structure affected by those nodes. We compared the performance of local updates with global updates of the structure in terms of query execution ratio and the communication overhead caused by the two types of updates. The proposed local updating mechanism is designed for coping well with such dynamic environments, by using distributed local link reversal. The simulation results demonstrate that geocasting with local updating performs a better query dissemination in terms of execution ratio with relatively low communication overhead, compared to geocasting with global updating. Although increasing number and speed of mobile nodes results in increase in the overall communication overhead on the network, local updating still performs better than global updating in terms of execution ratio. This is due to the fact that local updating performs continuous updating of the tree branches which are affected by the mobile nodes. The most critical issue is the mobility rate (*i.e.*, number of mobile nodes/total number of nodes) to decide which approach performs better. Indeed, if the network under consideration is highly mobile where most of the nodes are moving, tree based routing approaches become very costly for maintaining the routing structure. Although tree based routing protocols can tolerate topology changes in mostly static sensor network deployments, they cannot survive excessive topology changes in highly mobile deployments. For highly dynamic WSN applications, an alternative approach can be routing protocols that do not involve any pre-computation of distributed routing structures (e.g., trees) and give routing decision based on locally available information, e.g., [38].

## Figures and Tables

**Table 1.** Simulation Parameters.

Simulation time	100 *s*
Simulation area	1,800 × 1,800 *m*^2^
MAC protocol	IEEE 802.11 DCF
Transmission range	250 *m*
Number of sinks	3
Number of sensor nodes	60 to 360
Number of mobile nodes	10 to 50
Mobile node speed	1 to 17 *m/s* (4 to 60 *km/h*)
Mobility model	Random Waypoint
Global update frequency, *f*	3 *s* to 25 *s*
Mobile node beacon parameter, *k*	0.05, 0.10, 0.20
Target region	Circle, radius 250 *m*
Target region selection	5 random center points
Query sending frequency	10 *s* for local update simulations 100 *ms* before global updates
